# Cross-Cultural Adaptation, Reliability, and Validity of the Greek Version of the Upper Extremity Functional Index

**DOI:** 10.7759/cureus.33381

**Published:** 2023-01-05

**Authors:** Stefanos Karanasios, Georgios Kampourakis, Ilias Ntoulaveris, Kosmas Kouvaras, Ioannis Lignos, Nikolaos Diamantopoulos, George Gioftsos

**Affiliations:** 1 Physiotherapy, University of West Attica, Athens, GRC; 2 Physiotherapy, Hellenic Orthopedic Musculoskeletal Training (OMT) eDu, Athens, GRC; 3 Physiotherapy, Physio-Kifisia, Athens, GRC

**Keywords:** uefi, questionnaire, shoulder pain, translation, outcome measures, rotator cuff

## Abstract

Introduction

The Upper Extremity Functional Index (UEFI) is a region-specific questionnaire for patients with upper extremity disorders including patients with rotator cuff-related pain (RCRP). We aimed to translate and cross-culturally adapt the UEFI into Greek (UEFI-Gr) and evaluate its reliability and validity in a Greek-speaking population with RCRP.

Methods

Published guidelines for translation and cross-cultural adaptation of patient-rated outcome measures were followed. One hundred two patients were asked to complete the Greek versions of the UEFI; Disability of the Arm, Shoulder, and Hand (DASH) questionnaire; and RAND 36-Item Health Survey. Internal consistency, test-retest reliability, measurement error, content validity, concurrent validity, and ceiling and floor effects were evaluated.

Results

Minor linguistic discrepancies were identified and adopted in the Greek language. The UEFI-Gr presented high internal consistency (Cronbach’s alpha: 0.93), excellent test-retest reliability (intraclass correlation coefficient: 0.91; 95% confidence interval {CI}: 0.79-0.95), and acceptable measurement error (standard error of measurement: 4.9 points; minimal detectable change {MDC}: 13.8 points). No ceiling or floor effects were detected. Strong correlations were found with the Greek versions of the Disability of the Arm, Shoulder, and Hand questionnaire (r=0.629; p<0.001) and weak to moderate correlations with most subdomains of RAND 36-Item Health Survey (r=0.30-0.59; p<0.05).

Conclusions

The UEFI-Gr is a comprehensive, reliable, and valid self-reported instrument to evaluate symptoms in patients with RCRP. Further research on the responsiveness of the questionnaire is necessary.

## Introduction

Shoulder pain is considered the third most common complaint of all musculoskeletal conditions with rotator cuff-related pain (RCRP) contributing to over 70% of these cases [1-3]. Several pathoanatomical sources have been suggested to be related with RCRP including tendinopathy, bursitis, and/or partial or full-thickness tears of one or more of the tendons of the rotator cuff [1,4]. The clinical presentation of patients with RCRP includes shoulder pain and reduced range of motion with a significant impairment of their functional status and quality of life [4,5]. The accurate assessment of patients’ functioning is considered a fundamental step to provide prognosis, monitor the outcome, and guide the clinical decisions during the management of patients with RCRP [6,7]. Hence, using valid and reliable patient-reported outcome measures (PROMs) is strongly recommended to facilitate subjective evaluation of patients with the condition and provide accurate data in both research and clinical practice [8].

The Upper Extremity Functional Index (UEFI) is a region-specific PROM proposed to capture the multiple dimensions of patients’ functional status in various musculoskeletal conditions of the upper limb [9]. The UEFI includes 20 activities; each has a five-point rating scale (0: extreme difficulty; 4: no difficulty) that reflects the level of difficulty associated with each activity [9,10]. The possible score ranges between 0 and 80 with 0 indicating the lowest functional status and 80 indicating the highest functional status [9]. The original version of the questionnaire demonstrated excellent psychometric properties in terms of reliability (intraclass correlation coefficient {ICC}: 0.94), acceptable validity, and sensitivity to change (minimal clinically important difference: 8/80) in patients with upper extremity musculoskeletal disorders [11]. The UEFI has also been translated into three different languages such as Arabic, Turkish, and Chinese suggesting a reliable and valid outcome measure in patients with various upper extremity disorders including RCRP [12-14].

Cross-cultural adaptation and validation of PROMs in different languages are essential for making comparisons among patient groups with the same condition and promoting international research in the same field of interest [15]. The process of cross-cultural adaptation of PROMs requires a specific methodology aiming both the translation and the cultural adaptation in the new language in order to maintain the content validity of the PROM across different cultures [16].

Based on authors’ knowledge, a Greek version of the UEFI (UEFI-Gr) is not available yet. We hypothesized that a valid and reliable Greek version of the questionnaire will enable accurate monitoring of the efficacy of treatment procedures and facilitate clinical decision-making in patients with RCRP. The purpose of our study was to translate and cross-culturally adapt the English version of the UEFI into Greek and subsequently to examine its reliability and validity in patients with RCRP.

## Materials and methods

Cross-cultural adaptation

Prior to commencing the study, permission was obtained from the developer author (Prof. Paul W. Stratford). Translation and cross-cultural adaptation process followed a merged methodology of published guidelines [15-17].

Two bilingual independent translators (a nonmedical professional translator and a musculoskeletal physiotherapist) who were blinded to the construct of the scale completed the forward translation of the UEFI into Greek. The two Greek translations were synthesized into one by a research committee (two translators and three bilingual physiotherapists). Discrepancies were resolved through a consensus process, and when a conceptual equivalence was achieved, a final translation was produced. Two bilingual native English speakers (a research assistant and a physiotherapist), who were blinded to the scale, completed independently the back translation. The two versions of the back translation were carefully reviewed through a consensus process by the research committee including the author of the original version as well. During this step, the research committee decided if semantic, idiomatic, experimental, and conceptual equivalence between the English and Greek versions of the UEFI was reached [16].

To evaluate the comparability of language and similarity of interpretability of the pre-final version of the UEFI-Gr, we recruited seven bilingual physiotherapists who evaluated the pre-final version using a Likert scale (1: extremely comparable/similar; 7: not at all comparable/similar) [7]. For the cognitive debriefing of the pre-final version, seven healthy individuals (four females; mean age: 25.3 years) were recruited via personal contact. The members of the research committee considered carefully all provided comments resulting in the pre-final version of the questionnaire.

To assess the content validity (relevance) of the questionnaire, five physiotherapy researchers with expertise in the current field were asked to judge each item using a four-point Likert scale (1: not relevant and clear; 4: highly relevant and clear) [18]. Also, face validity and comprehensibility of the questionnaire were assessed by administrating the pre-final version in 10 patients with RCRP (five males and five females; mean age±SD years: 43.8±6.93; range: 32-56). Participants were asked about the clarity of the terms, instructions, and response options of the questionnaire. Also, they were asked to evaluate the comprehensiveness of the scale and the relevance of the items included regarding their condition. After completing pilot testing, the final version of the UEFI-Gr was reached.

Patients

We recruited individuals with RCRP from various hospitals and outpatient rehabilitation practices in Greece between April and December 2022. Patients were assessed for eligibility by a physiotherapist with 18 years of experience in musculoskeletal conditions. Participants were males and females, older than 18 years old, and fluent in the Greek language. The exclusion criteria were as follows: not able to read Greek; age of less than 18 years old; suffering from neck, thoracic, or, other than the shoulder, upper extremity problems affecting their shoulder function; chronic inflammatory diseases such as rheumatoid arthritis; fracture; cancer; central nervous system disorders; and cognitive disability.

Procedures

Prior to entering the study, all patients signed an informed consent form. At the initial visit, the participants’ demographic characteristics were recorded. The study was approved by the University of West Attica Ethics Committee (41370/18-04-2022).

To assess convergent validity, participants were asked to complete the Greek versions of the UEFI; Disability of the Arm, Shoulder, and Hand (DASH) questionnaire; and RAND 36-Item Health Survey in a quiet place without any help. The DASH questionnaire has been previously used to validate the UEFI in different languages [12-14]. It includes 30 items, and its item is scored with a five-point scale based on the level of difficulty [19]. The final score fluctuates between 0 and 100 (0: no disability; 100: the highest disability) [20]. The RAND 36-Item Health Survey is a generic questionnaire for the quality of life and laps eight concepts: physical functioning, bodily pain, role limitations due to physical health problems, role limitations due to personal or emotional problems, emotional well-being, social functioning, energy/fatigue, and general health perceptions. It has shown excellent test-retest reliability and poor to moderate correlation with DASH in patients with shoulder pain [7].

To assess the reliability of UEFI-Gr, participants were given a second copy of the questionnaire and were asked to fill and return it after 2-7 days. the data of the second UEFI-Gr was used only if the patients’ condition was considered unchanged in terms of symptoms and function from baseline.

Statistical analysis

Based on COSMIN recommendations, a minimum sample size of 100 participants was considered adequate for the purposes of the validation of the study [13,21]. For the evaluation of test-retest reliability and measurement error, a minimum sample size of 50 participants were considered good to excellent [13,21]. Parametric tests were used after using the Shapiro-Wilk test and Q-Q plots to ensure that the data was normally distributed. The participants’ characteristics and outcome measures were presented using descriptive statistics. Statistical Package for Social Sciences (SPSS) (IBM SPSS Statistics, Armonk, NY) was used for data analysis. The statistically significant level was set at p<0.05.

Five experts and 10 patients with RCRP were recruited to judge item-content relevance. We have used Aiken’s coefficient (V) to analyze each item’s content validity (values of >0.70 were considered with acceptable validity) [22].

To assess internal consistency, we used Cronbach’s alpha with values of >0.70 considered of good internal consistency [17]. To assess test-retest reliability, the ICC (two-way random model and absolute agreement) with 95% confidence interval (CI) was used. ICC values of >0.75, from 0.4 to 0.75, and <0.4 were considered excellent, fair, and poor, respectively [23]. Absolute reliability was analyzed using the standard error of measurement (SEM=SD×($\sqrt{1-testretest reliability coefficient }$)) and minimal detectable change (MDC) (MDC_95_=1.96×$\sqrt{2 }$×SEM).

We analyzed the content validity index (CVI) by calculating the items rated >3 divided by the number of experts. We assumed that item-CVI>0.83 and scale-CVI>0.80 correspond to acceptable values [18].

Convergent validity was evaluated using Pearson’s correlation coefficient (r). The UEFI-Gr score (first administration) and the scores of the Greek versions of DASH questionnaire and RAND 36-Item Health Survey were used. Pearson’s correlation coefficient values of ≥0.60, between 0.40 and 0.59, and ≤0.39 were considered as strong, moderate, and weak correlation between the questionnaires, respectively [13]. We hypothesized a strong correlation between UEFI-GR and the Greek versions of DASH and a poor correlation with the RAND 36-Item Health Survey subscales as it was confirmed in other versions [11].

Time to complete the UEFI-Gr was also recorded. Further, floor and ceiling effects were estimated when more than 15% of all responders scored the minimum (0) or the maximum (80) possible score.

## Results

Translation, cross-cultural adaptation, and item content validity

Three linguistic discrepancies in items were identified and resolved by consensus during forward and backward translation (Table 1). Five experts and 10 patients with RCRP were interviewed for comprehensibility, reporting no issues regarding the wording of the items resulting in the final version of the UEFI-Gr.

**Table 1:** List of items that were adjusted during cross-cultural translation

Items requiring adjustment	Rationale	Solutions
“Extreme”	Literal translation was not considered appropriate	Instead, the word “maximum” translated into Greek was used
“Pushing up on your hands”	Literal translation was not available	“Lifting the body” was used keeping the same meaning in Greek
“Lacing shoes”	Literal translation was not available	The expression “placing the laces on the shoes” was described in Greek

Participants

A total of 102 patients (43 females and 59 males) with a mean age (±SD) of 48.7 (±16.1) years were included in the study. Table 2 shows the participants’ demographic characteristics. The responders required 4-5 minutes to complete the questionnaire.

**Table 2:** Demographic and clinical characteristics of participants (N=102) DASH, Disability of the Arm, Shoulder, and Hand; UEFI-Gr, Upper Extremity Functional Index in Greek; N, number of samples

Characteristic	Mean±SD (range) or number (percentage)
Age (years)	48.7±16.1 (18-84)
Sex	
Males	59 (57.8%)
Females	43 (42.2%)
Dominant side	
Right	78 (76.4%)
Left	24 (23.5%)
Affected side	
Right	64 (62.7%)
Left	38 (37.3%)
Height (cm)	170.6±8.6 (152-192)
Weight (kg)	76.3±11.9 (47-102)
UEFI-Gr (points)	60.8±19.7 (48.6±15.7)
DASH	41.2±18.4
RAND-36	
Physical functioning	67.7±18.5
Physical health	39.6±37.6
Emotional problems	57.5±40.9
Energy/fatigue	48.1±22.5
Emotional well-being	60.7±23.4
Social functioning	69.01±22.4
Pain	49.02±24
General health	59.8±15.8
Health change	59.8±21.6

Reliability

Two patients reported a substantial improvement between the first and second administration of the questionnaire and were excluded from the analysis. Seventy-three patients were finally included in the reliability analysis suggesting an excellent test-retest reliability (ICC: 0.91; 95% CI: 0.79-0.95) and a high internal consistency of the questionnaire (Cronbach’s alpha: 0.93) (Table 3). The standard error of mean (SEM) and MDC_95_ were 4.9 and 13.8 points (scales 0-80), respectively.

**Table 3:** Measurement properties of translated UEFI versions *Non-statistically significant correlations (p>0.05) ICC, intraclass correlation coefficient; UEFI, Upper Extremity Functional Index; DASH, Disability of the Arm, Shoulder, and Hand; SEM, standard error of mean; MDC, minimal detectable change; MCID, minimal clinically important difference; PF, physical functioning; PH, physical health; EP, emotional problems; E/F, energy/fatigue; EWB, emotional well-being; SF, social functioning; P, pain; GH, general health; HC, health change; SPADI, Shoulder Pain and Disability Index; NPRS, Numerical Pain Rating Scale; GAF, global assessment of function; SF-36, short form-36; PLS, pain limitation scale; PIS, pain intensity scale; UEFS, Upper Extremity Functional Scale; CI: confidence interval; N, number of samples

	Greek (N=102)	English (N=255)	Arabic (N=109)	Turkish (N=93)	Chinese (N=25)
Reproducibility ICC (95% CI)	0.91 (0.79-0.95)	0.94 (0.93-0.96)	0.92 (0.85-0.96)	0.80 (0.60-0.90)	0.97
Internal consistency (Cronbach’s alpha)	0.93	0.94	0.96	0.89	0.93
Measurement error (out of 80 points)	SEM: 4.9; MDC_95_: 13.8	SEM: 4; MDC_90_: 9.4	SEM: 5.5; MDC_90_: 12.8	-	-
Convergent validity	-0.62 (DASH)	0.54 (PLS)	-0.95 (DASH)	-0.61 (SPADI)	-0.86 (DASH)
	0.33 (RAND-36 PF)	0.44 (PIS)	-0.48 (NPRS)		
	0.47 (RAND-36 PH)	0.95 (UEFI-15)	0.56 (GAF)	-0.63 (quick-DASH)	
	0.30 (RAND-36 EP)	0.54 (PLS)	0.77 (RAND-36 PF)		
	0.31 (RAND-36 E/F)	0.44 (PIS)	0.60 (RAND-36 PH)	-0.05 (SF-36)	
	0.18 (RAND-36 EWB)*	0.95 (UEFI-15)	0.69 (RAND-36 P)		
	0.38 (RAND-36 SF)	0.54 (PLS)	0.47 (RAND-36 EWB)		
	0.59 (RAND-36 P)		-0.95 (DASH)		
	0.17 (RAND-36 GH)*		-0.48 (NPRS)		
	0.23 (RAND-36 HC)		0.56 (GAF)		
Ceiling and floor effects	0 and 0	0 and 0	0 and 0	0 and 0	-

The item-CVI was found between 0.93 and 1.00, the scale-CVI/universal agreement was 0.91, and scale-CVI/average was 0.973. There was a strong negative correlation between the Greek versions of UEFI and DASH (r=-0.629; p<0.001) (Table 3). A significant positive correlation was found between UEFI-GR and most subdomains of the RAND-36 suggesting a weak to moderate correlation (Table 3). There were no ceiling and floor effects of the questionnaire.

## Discussion

Based on the study findings, the UEFI was successfully translated and cross-cultural adapted into Greek. The psychometric properties of the questionnaire were similar to other translated versions (Table 3). The UEFI-Gr showed adequate face and content validity and a strong negative correlation compared to DASH. Also, the Greek version of the questionnaire presented an excellent test-retest reliability with an acceptable measurement error.

For the translation and cross-cultural adaptation process, we adopted a rigorous approach using a merged methodology from published guidelines [16,17,24,25]. We have found some linguistic discrepancies between the original and the Greek translation (items c, d, and o), which were appropriately resolved by the research committee. Similar discrepancies were reported in the Arabic (item r) and Turkish versions (items c, d, and e), which also included a systematic approach to semantic, idiomatic, experiential, and conceptual equivalences between the original and the targeted version [12,13].

The internal consistency of the UEFI-Gr was high (Cronbach’s alpha: 0.93) and similar to the original and other translated versions (Cronbach’s alpha: 0.89-0.96) (Table 3). In the same vein, UEFI-Gr showed an excellent test-retest reliability (ICC: 0.91), which was within the range of other published versions (ICC: 0.80-0.97) (Table 3). Accordingly, the reported measurement error was similar to previous evaluations (SEM: 4.8 points; MDC_95_: 13.8 points). Only the Turkish version presented a lower test-retest reliability (ICC: 0.80; 95% CI: 0.60-0.90), which can be partially attributed to the lower sample size (35 participants) used for the reliability analysis compared to the other studies (Table 3) [13]. Another explanation may be based on the time course between test-retest measurements, which was not reported in the Turkish version. The time interval between test-retest evaluation is considered a critical factor in reliability evaluations [24]. The available UEFI translations with higher ICC values (i.e., Arabic, Chinese, and Greek versions) were included less than a week between the administrations. We also used a time interval of between two and seven days to ensure that the patients’ condition have not changed. Also, we aimed to reduce the possible risk of systematic bias in reliability analysis by evaluating the stability of the patients’ condition. As a result, some responses were excluded from statistical analysis due to significant changes. It should be noted that the relationship between time interval, response options, patient condition, and recall bias remains unclear and difficult to control in test-retest reliability analysis [26].

To assess convergent validity, we decided to use another region-specific questionnaire for the upper limb such as the DASH questionnaire. Our correlation results regarding the DASH questionnaire were consistent with previous reports (0.63-0.95) confirming the strong association between the two PROMs. The correlations were found strong suggesting that the UEFI-Gr is a valid tool to assess shoulder function in patients with RCRP; however, they were not perfect and substantially differed between studies. A possible explanation is that both questionnaires as region-specific instruments include a wide range of functional impairments. Another reason may lie on the sample characteristics among the translated versions that consisted of patients with RCRP and various upper extremity musculoskeletal disorders or trauma [12-14]. As was expected, the correlations between UEFI-Gr and RAND-36 subscales were poor to moderate and in agreement to previous reports of UEFI versions. This finding can be attributed to the construct of RAND-36 subscales, which are designed to measure domains that are too generic or irrelevant to shoulder pain (Table 3).

Limitations and future research

Although an optimal time period of 7-14 days is widely accepted for test-retest reliability in musculoskeletal research, we decided to use a short time interval (2-7 days) to ensure stability of the patients’ condition. However, the short time interval may have significantly increased the risk of recall bias in our study [27]. Our study included patients with RCRP and cannot be generalized to other patient groups. Therefore, we suggest that further research should investigate the psychometric properties of the UEFI-Gr in patients with various musculoskeletal disorders of the upper limb. Responsiveness is another critical psychometric property, which illustrates the ability of a scale to detect clinically important changes over time, even if these changes are small [17]; nevertheless, our study did not include the evaluation of this important property. Hence, further research focusing on responsiveness is necessary for the use of the UEFI-Gr version in the clinical setting.

## Conclusions

The Greek version of the UEFI presented a high internal consistency, excellent test-retest reliability, and strong negative correlation with a well-investigated and validated outcome measure such as the DASH questionnaire. Based on the present findings, the UEFI-Gr can be suggested as a standard outcome measure in Greek-speaking patients with RCRP. Further evaluation of the responsiveness of the PROM is essential.

## References

[1] Littlewood C, May S, Walters SJ (2013). A review of systematic reviews of the effectiveness of conservative interventions for rotator cuff tendinopathy. Shoulder Elbow.

[2] Murphy RJ, Carr AJ (2010). Shoulder pain. BMJ Clin Evid.

[3] Britt H, Miller GC, Henderson J (2016). General practice activity in Australia 2015-16. http://hdl.handle.net/2123/15514.

[4] MacDermid JC, Ramos J, Drosdowech D, Faber K, Patterson S (2004). The impact of rotator cuff pathology on isometric and isokinetic strength, function, and quality of life. J Shoulder Elbow Surg.

[5] Ackerman IN, Fotis K, Pearson L (2022). Impaired health-related quality of life, psychological distress, and productivity loss in younger people with persistent shoulder pain: a cross-sectional analysis. Disabil Rehabil.

[6] Furtado R, MacDermid JC, Nazari G, Bryant DM, Faber KJ, Athwal GS (2020). Cross-cultural adaptions and measurement properties of the WORC (Western Ontario rotator cuff index): a systematic review. Health Qual Life Outcomes.

[7] Karanasios S, Korakakis V, Diochnou A, Oikonomou G, Gedikoglou IA, Gioftsos G (2022). Cross cultural adaptation and validation of the Greek version of the Western Ontario rotator cuff (WORC) index. Disabil Rehabil.

[8] Furtado R, Bobos P, Ziebart C, Vincent J, MacDermid J (2022). Patient-reported outcome measures used for shoulder disorders: an overview of systematic reviews. J Hand Ther.

[9] Hamilton CB, Chesworth BM (2013). A Rasch-validated version of the upper extremity functional index for interval-level measurement of upper extremity function. Phys Ther.

[10] Stratford PW (2001). Development and initial validation of the upper extremity functional index. Physiother Can.

[11] Chesworth BM, Hamilton CB, Walton DM (2014). Reliability and validity of two versions of the upper extremity functional index. Physiother Can.

[12] Aljathlani MF, Alshammari MO, Alsuwaygh MA, Al-Mutairi MS, Aljassir FF, Bindawas SM, Alnahdi AH (2022). Cross-cultural adaptation and validation of the Arabic version of the upper extremity functional index. Disabil Rehabil.

[13] Aytar A, Yuruk ZO, Tuzun EH, Baltaci G, Karatas M, Eker L (2015). The upper extremity functional index (UEFI): cross-cultural adaptation, reliability, and validity of the Turkish version. J Back Musculoskelet Rehabil.

[14] Xiao X, Yang Z, Xia X (2012). The reliability and validity of the Chinese version of the upper extremity functional index. Chin J Phys Med Rehabil.

[15] Wild D, Grove A, Martin M, Eremenco S, McElroy S, Verjee-Lorenz A, Erikson P (2005). Principles of good practice for the translation and cultural adaptation process for patient-reported outcomes (PRO) measures: report of the ISPOR Task Force for translation and cultural adaptation. Value Health.

[16] Beaton DE, Bombardier C, Guillemin F, Ferraz MB (2000). Guidelines for the process of cross-cultural adaptation of self-report measures. Spine (Phila Pa 1976).

[17] Terwee CB, Bot SD, de Boer MR (2007). Quality criteria were proposed for measurement properties of health status questionnaires. J Clin Epidemiol.

[18] Polit DF, Beck CT, Owen SV (2007). Is the CVI an acceptable indicator of content validity? Appraisal and recommendations. Res Nurs Health.

[19] Themistocleous GS, Goudelis G, Kyrou I (2006). Translation into Greek, cross-cultural adaptation and validation of the disabilities of the arm, shoulder, and hand questionnaire (DASH). J Hand Ther.

[20] Hudak PL, Amadio PC, Bombardier C (1996). Development of an upper extremity outcome measure: the DASH (disabilities of the arm, shoulder and hand) [corrected]. The Upper Extremity Collaborative Group (UECG). Am J Ind Med.

[21] Terwee CB, Mokkink LB, Knol DL, Ostelo RW, Bouter LM, de Vet HC (2012). Rating the methodological quality in systematic reviews of studies on measurement properties: a scoring system for the COSMIN checklist. Qual Life Res.

[22] Aiken LR (1985). Three coefficients for analyzing the reliability and validity of ratings. Educ Psychol Meas.

[23] Cicchetti DV (1994). Guidelines, criteria, and rules of thumb for evaluating normed and standardized assessment instruments in psychology. Psychol Assess.

[24] Sperber AD (2004). Translation and validation of study instruments for cross-cultural research. Gastroenterology.

[25] Prinsen CA, Mokkink LB, Bouter LM, Alonso J, Patrick DL, de Vet HC, Terwee CB (2018). COSMIN guideline for systematic reviews of patient-reported outcome measures. Qual Life Res.

[26] Korakakis V, Saretsky M, Whiteley R (2019). Translation into modern standard Arabic, cross-cultural adaptation and psychometric properties' evaluation of the lower extremity functional scale (LEFS) in Arabic-speaking athletes with anterior cruciate ligament (ACL) injury. PLoS One.

[27] Marx R, Menezes A, Horovitz L, Jones E, Warren R (2003). A comparison of two time intervals for test-retest reliability of health status instruments. J Clin Epidemiol.

